# Electrical Characterisation of Aδ-Fibres Based on Human *in vivo* Electrostimulation Threshold

**DOI:** 10.3389/fnins.2020.588056

**Published:** 2021-01-05

**Authors:** Shota Tanaka, Jose Gomez-Tames, Toshiaki Wasaka, Koji Inui, Shoogo Ueno, Akimasa Hirata

**Affiliations:** ^1^Department of Electrical and Mechanical Engineering, Nagoya Institute of Technology, Nagoya, Japan; ^2^Center of Biomedical Physics and Information Technology, Nagoya Institute of Technology, Nagoya, Japan; ^3^Department of Functioning and Disability, Institute for Developmental Research, Aichi Developmental Disability Center, Kasugai, Japan; ^4^Department of Integrative Physiology, National Institute for Physiological Sciences, Okazaki, Japan; ^5^Department of Biomedical Engineering, Graduate School of Medicine, The University of Tokyo, Tokyo, Japan; ^6^Frontier Research Institute for Information Science, Nagoya Institute of Technology, Nagoya, Japan

**Keywords:** strength duration curve, selective stimulation of small fibres, extracellular electric field, electrical analysis in biological tissues, electrical stimulation

## Abstract

Electrical stimulation of small fibres is gaining attention in the diagnosis of peripheral neuropathies, such as diabetes mellitus, and pain research. However, it is still challenging to characterise the electrical characteristics of axons in small fibres (Aδ and C fibres). In particular, *in vitro* measurement for human Aδ-fibre is difficult due to the presence of myelin and ethical reason. In this study, we investigate the *in vivo* electrical characteristics of the human Aδ-fibre to derive strength–duration (S–D) curves from the measurement. The Aδ-fibres are stimulated using coaxial planar electrodes with intraepidermal needle tip. For human volunteer experiments, the S–D curve of Aδ-fibre is obtained in terms of injected electrical current. With the computational analysis, the standard deviation of the S–D curve is mostly attributed to the thickness of the stratum corneum and depth of the needle tip, in addition to the fibre thickness. Then, we derive electrical parameters of the axon in the Aδ-fibre based on a conventional fibre model. The parameters derived here would be important in exploring the optimal stimulation condition of Aδ-fibres.

## Introduction

Different receptors and peripheral nerve fibres convey somatosensory information to the central nervous system. Selective stimulation of nerve fibres is essential in observing the activation of a somatosensory submodality system. The rationale for this is based on different conduction velocities of fibres with different thicknesses (Vallbo et al., 1979). The brain responses for first arriving signals may suppress the effect of those for later arriving signals.

Terminals of afferent Aδ- and C-fibres are located in the epidermis. Meanwhile, mechanoreceptors are located in the upper layer of the dermis. Unlike the activation of the touch system, which is characterised by the stimulation of thick Aβ fibres, thin Aδ- or C-fibres can be stimulated in a selective manner for pain system activation. Recent studies used selective stimulation of small fibres in patients suffering from peripheral neuropathies, such as diabetes mellitus (Kukidome et al., 2015; Omori et al., 2017; Suzuki et al., 2020).

There are several techniques for selective stimulation of thin fibres based on the electrophysiological differences among peripheral fibres: radiant heat stimulation by lasers for Aδ-fibres (Bromm et al., 1983; Bragard et al., 1996; Magerl et al., 1999; Opsommer et al., 2001; Nahra and Plaghki, 2003; Churyukanov et al., 2012) and intraepidermal electrical stimulation (IES) for both C- and Aδ-fibres (Inui et al., 2002).

IES uses a small concentric bipolar needle electrode to inject a current of a few milliamperes to generate a focused electric field around the electrodes (Inui and Kakigi, 2012). Different stimulation conditions have been proposed in applying selective stimulation of Aδ- and C-fibres in different studies: different polarities of electrode, durations, larger interstimulus interval, and waveforms (Inui and Kakigi, 2012; Kodaira et al., 2014; Hugosdottir et al., 2019). The stimulation of Aδ- and C-fibres has been determined based on the latency in the fibres. Thus, optimal stimulation condition for these small fibres is a challenging topic. Except for the diameter, the physical parameters of the fibres are still unknown and controversial (Reilly and Diamant, 2011).

One method used to characterise the fibre response and its electrical characteristics is the strength–duration relationship (S–D curve), which represents the threshold dependence of the pulse duration for electrostimulation. The electrostimulation threshold becomes small with the increase in the pulse durations until converging to a minimum threshold termed “rheobase.” Only two studies have measured perception threshold to derive S–D curves but were not specific to one type of small fibre (co-activation of different small fibres) (Hennings et al., 2017). Recently, Poulsen et al. (2020) investigated S–D curves of Aδ-fibres for different electrodes; the rheobase was estimated in terms of injection current, which is not induced by physical quantity. Moreover, only a few pulse durations were considered. All these experiments are based on *in vivo* experiments and assume activation thresholds of the fibre equal to activation perception sensation in the experiment. This is because they have not yet succeeded in creating the human Aδ-fibres for *in vitro* experiment, because of the presence of myelin. Thus, the rheobase of Aδ-fibres has not been well quantified, except for an empirical estimation (Reilly and Diamant, 2011).

Computational electromagnetics has become a powerful tool in determining the induced electric field for an external field exposure or current injection. To date, only a few studies have been performed using electromagnetic dosimetry in peripheral nerves in the skin (Mørch et al., 2011; Frahm et al., 2013; Hirata et al., 2013a). In our previous study (Motogi et al., 2016), we conducted computational dosimetry of the skin for the injection current to assess nerve stimulation.

The present study aimed to estimate the electrical parameters of Aδ-fibres via comparison of *in vivo* experiment and computation. One of the features of this study is that the computation is based on multiscale stimulation combining electromagnetic dosimetry and nerve activation modelling.

## Materials and Methods

### IES Experiment

**FIGURE 1:**
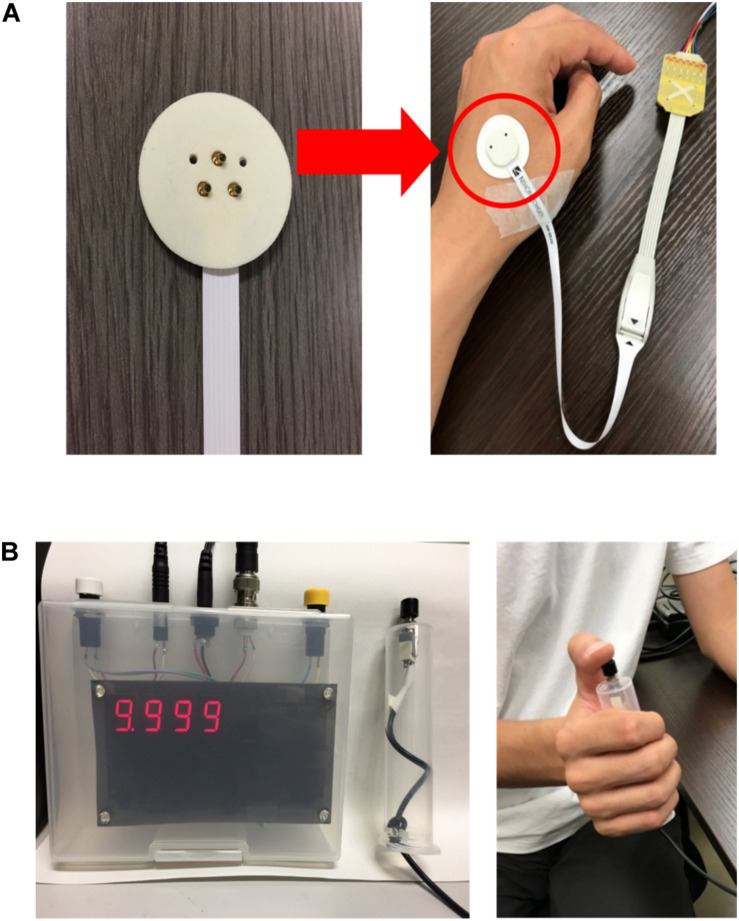
Experimental setup: **(A)** electrode configuration and location on the hand and **(B)** reaction time were automatically measured and displayed (left) after the subject pressed a push button (right) during the perception threshold detection experiment.

The experiments in measuring perception threshold through Aδ-fibre stimulation were performed on 14 healthy male subjects (age, 21.8 ± 1.7 years). The experiments were approved by the Ethical Committee of the Nagoya Institute of Technology. The experimental setup is presented in Figure 1. The stimulation device (STG4004, Multi Channel Systems GmBH, Germany) delivered a single square pulse current through a concentric bipolar needle electrode (NM-983 W, Nihon Kohden, Tokyo, Japan). The inner needle and ring electrodes were assigned to the anode and cathode of the stimulation device, respectively. The pulse duration and current intensity were varied during the experiment to explore the threshold. The stimulation was applied to the dorsum of the left hand.

**FIGURE 2:**
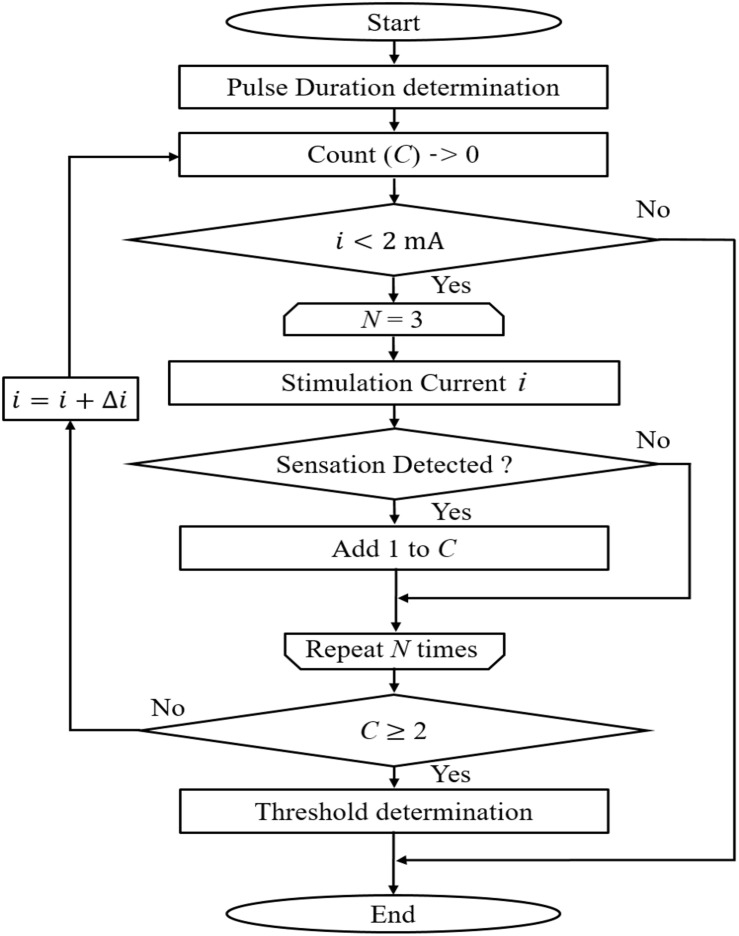
Experimental protocol for perception threshold detection in ascending trial (descending is not shown). In this study, *N* was set at 3.

The experimental protocol to measure the perception threshold (defined as the lowest injection current at which the subject feels a sensation) is shown in Figure 2. The subjects were instructed to press a button as soon as they felt the minimum sensation. The time between stimulation onset and detection by pressing a button (termed reaction time) was automatically recorded. The method of limits was used to determine the stimulus threshold with consecutive ascending and descending trials. A total of six trials were performed per subject. For ascending trials, the injection current intensity was increased in steps of 0.05 mA. Each session of the ascending trial consisted of *N* consecutive stimulations with the same current intensity and fixed stimulation duration, in which the stimulations were delivered at an undisclosed time to the subject. We set *N* to 3 to obtain a reliable perception threshold in a set. The threshold was considered detected when the subject reported a sensation at least two of three times with reaction time in the range of Aδ-fibre transmission (200–800 ms) (Ragé et al., 2010; Kodaira et al., 2014). The rest time between sets of three stimulations was 1 min to reduce the effect of habituation. If a maximum limit of 2 mA was reached, the perception threshold was considered as not found. For descending trials, the current intensity was decreased by 0.05 mA until the stimulus was not detected. The mean of the six trials was obtained as the stimulus threshold. Six sessions were conducted for different pulse durations (60, 100, 200, 400, 800, and 1600 μs), which were randomly selected. The rest time between three stimulation sets was also 1 min to reduce the effect of habituation.

### Modelling Needle Electrodes and the Skin

**FIGURE 3:**
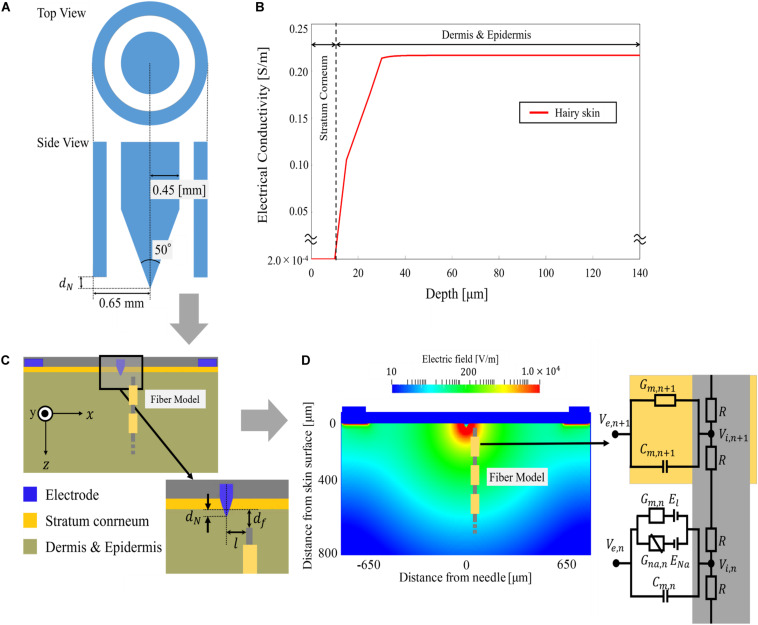
Multiscale intraepidermal electrical stimulation model: **(A)** intraepidermal electrical stimulation electrode, **(B)** profile of electrical conductivity estimated from the water content of the tissue, **(C)** layered skin model with assumption of the position of a single Aδ-fibre, and **(D)** the electric field distribution on the transversal plane for an injection current of 0.02 mA with an equivalent cable model of Aδ-fibre.

Our IES model was almost identical to that in our previous study (Motogi et al., 2016). In brief, the concentric bipolar needle electrode used in the experiment is modelled as shown in Figure 3A (Motogi et al., 2014). The inner electrode and ring electrode corresponded to the cathode and anode, respectively, and modelled as a perfect conductor. The part of the inner needle protruding from the outer ring (called *d*_*N*_ ‘needle depth’ hereafter) was selected between 5 to 30 μm for experiments mentioned in section “Computational Estimation of Electric Field in the Skin Model” and fixed to 20 μm in section “Development of Computational Aδ-Fibre Model”.

The skin was modelled as a layered structure for hairy skin (Alekseev and Ziskin, 2007; Schmid et al., 2013; De Santis et al., 2015; Motogi et al., 2016). The thickness of the stratum corneum layer was 10–20 μm. The boundary between the epidermis and dermis layers was treated as a transition region (10–30 μm). The epidermis thickness was reported as 50–70 μm (Valentin, 2002; Koster et al., 2012). The corresponding conductivities values are shown in Figure 3B. In detail, the conductivity of the stratum corneum was selected as 2 × 10^–4^ [S/m] at 2.1 kHz based on Gabriel et al. (1996). In the transit region between the epidermis and dermis, the variation of the conductivity was approximately proportional to the water content (Faes et al., 1999; Egawa and Kajikawa, 2009). The conductivity of the dermis at sufficient depth was 0.21 S/m based on Sasaki et al. (2016).

The dimensions of the skin model were 1.54 mm (depth) × 1.65 mm × 1.65 mm and discretised by voxels of 5 μm of length. We confirmed no variation of the electric field if larger dimensions are used as the current flow is confined between the electrodes.

### Volume Conductor Model of Skin

The electrical skin model is treated as a passive volume conductor to compute the *in situ* electric field produced by the injected current considering that the frequency is below kilohertz range and displacement current is negligible (Hirata et al., 2013b). The effects of the electric fields generated by neural activity was ignored.

The scalar potential finite difference method (Dawson and Stuchly, 1996) is used to numerically solve the following equation to obtain the scalar potential ϕ:

(1) ∇⁡(σ∇⁡(φ))

where σ denotes the tissue conductivity. The potential was solved iteratively using the successive-over-relaxation and multigrid methods (Laakso and Hirata, 2012). A current source was connected between the inner electrode and outer ring of the electrode. To obtain the *in situ* electric field, the potential difference between nodes of the voxel was divided by the voxel length. The injection current to generate an electric field distribution that can depolarise a fibre and generate an action potential was computed in the following section.

### Nerve Activation Modelling for Aδ-fibre

The effects of the extracellular electric field derived from Eq. (1) on a myelinated nerve fibres were described using the following general equation (McNeal, 1976; Rattay, 1999):

(2) $$\begin{array}{c} C_{m,n}\frac{dV_{m,n}}{dt}+I_{ion,n}-\frac{V_{m,n-1}-2V_{m,n}-2V_{m,n+1}}{0.5\left(R_{m,i}+R_{m,n}\right)}\\ \;=\frac{V_{e,n-1}-2V_{e,n}-2V_{e,n+1}}{0.5\left(R_{m,i}+R_{m,n}\right)} \end{array}$$

where *C*_*m*_ is the membrane capacitance and *R*_*m,i*_ and *R*_*m,n*_ are the internode membrane resistivity and nodal membrane resistivity, respectively. The membrane potential is represented by the variable *V*_*m,n*_ = *V*_*e*_ – *V*_*i*_. The fibres are formed by internodes (myelin segments) and nodes of Ranvier (ionic channels) as shown in Figure 3D. At the internodes, the membrane current *I*_*ion,n*_ was modelled by the passive conductance (*G*_*m,n*_) multiplied by the membrane potential. At the nodes of Ranvier, the ionic membrane current was formulated using the Chiu–Ritchie–Rogart–Stagg–Sweeney model (CRRSS), which is a conductance-based voltage-gated model (Sweeney et al., 1987). Here, the ionic current follows

(3) $$I_{ion,n}=G_{Na}m^{3}h\left(V_{m,n}-E_{Na}\right)+G_{L}\left(V_{m,n}-E_{L}\right)$$

where *G*_*L*_ and *G*_*Na*_ are the leak passive conductance and sodium channel conductance, respectively, and *E*_*Na*_ and *E*_*L*_ are the reversal potentials. Sodium channel activation and inactivation are controlled by the dimensionless variables *m* and *h*.

The smallest injection current to propagate an action potential in an axon was obtained (called activation threshold) using a search method (bisection method) (Gomez-Tames et al., 2018) until the error was smaller than 10 μA. An action potential was elicited when the membrane potential was depolarised up to 80 mV in at least four neighbouring nodes at successive times (Reilly, 2016). It is reasonable to apply a similar algorithm to central nervous tissue when the target is direct simulation of pyramidal tracts.

### S–D Curve

The S–D curve is the relationship between the activation threshold and pulse duration. Two metrics were derived for the S–D curve: rheobase and chronaxie. However, unlike *in vitro* studies, we defined threshold as the perception threshold instead of activation threshold. Because the perception was caused by synaptic mechanism of the peripheral and central nervous systems, actual threshold of activation of a single fibre would be smaller than that reported in this study.

Rheobase is the minimum current intensity that elicits an activation that occurs after a long duration, and chronaxie is the time when stimulation current is twice the rheobase. The mathematical expression for the S–D curve was as follows:

(4) I=b(1+C/w)

where *I* is the input current, *b* is the rheobase, *c* is the chronaxie, and *w* is the pulse width. These parameters described the excitability characteristics of different nerve fibres. The effect of electrical parameters in the circuit model on rheobase and chronaxie was examined.

### Nerve Parameters

The Aδ-fibre diameter (*D*) was in the range of 0.68–1.46 μm, with an average of 1.08 ± 0.15 μm (Reilly et al., 1997). The depth from the skin surface (*d*_*f*_) of Aδ-fibre was between 10 and 40 μm (MacIver and Tanelian, 1993). In this study, to fit the experimental S–D curve, the diameter of the fibre model was fixed to *D* = 1.0 μm and *d*_*f*_ = 25 μm (Figure 3C).

The nerve fibre model for activation was based on the CRRSS model, and then the parameters in the model were explored. The electrical parameters of the CRRSS model are shown in the Supplementary Table 1. The fibre model was initially placed at *l* = 0.25, 0.30, and 0.35 mm. This was because the subjects could feel the sensation originated when multiple nerves were stimulated and integrated. The experimental values were considered to be the thresholds of the nerve located farthest from the electrode among multiple nerves that were stimulated. Second, the *C* and *G*_*l*_ parameters were modified from the original values to adjust the chronaxie value (Sweeney et al., 1987). We adopted the least squares error for each pulse width to adjust the chronaxie. The electrical parameters used in the modified CRRSS model are listed in Table 1.

**TABLE 1:** Coefficient of variation in measured results and its normalised value by rheobase.

Pulse width (μ s)	Coefficient of variation	
	Non-normalised	Normalised
60	0.30	0.37
100	0.36	0.34
200	0.42	0.37
400	0.38	0.22
800	0.50	0.16
1600	0.33	0.18

Note that these fibres and electrode/skin spatial parameters are closely related to each other and thus should be fitted in an iterative manner. However, for simplicity, the spatial distance *l* was fixed, as mentioned above, and then the remaining fibre parameters are shown. The presented results are results of three iterations.

## Experimental and Computational Results

### Experiment Results of Aδ-Fibre

**FIGURE 4:**
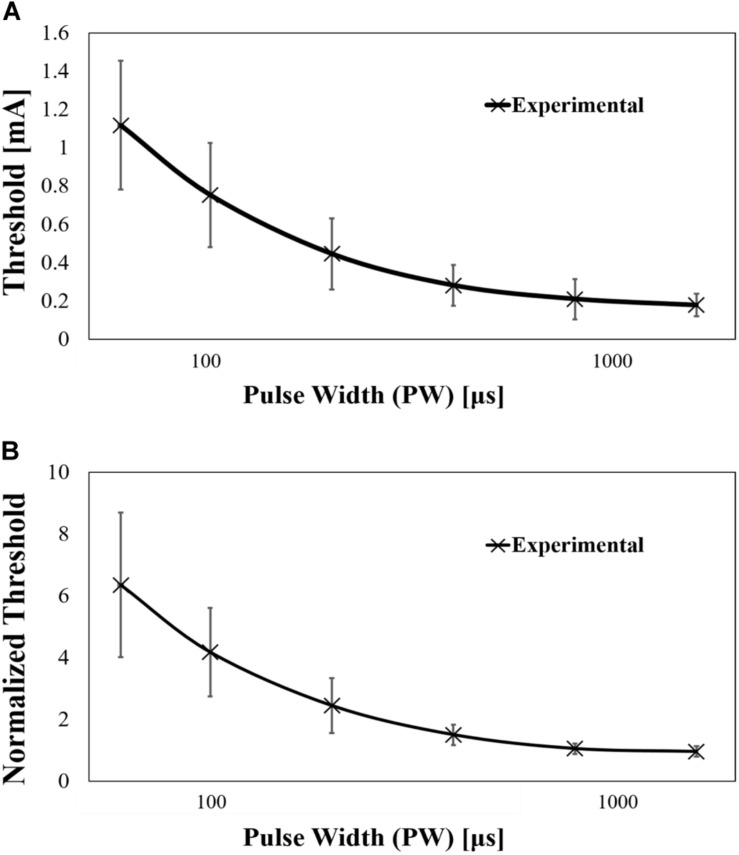
**(A)** Experimental strength–duration curves of Aδ-fibre by single-pulse stimulation (mean value and standard deviation, *n* = 14) and **(B)** its normalisation by rheobase (mean value).

The current amplitude of Aδ-fibre for a single pulse injection was obtained experimentally, as shown in Figure 4A. The mean rheobase and chronaxie were 0.178 mA [standard deviation (SD) of 0.057 mA] and 270 μs (SD of 120 μs), respectively. As will be discussed later, a part of the variability of the rheobase was attributed to the morphology of the skin and depth of the needle tip. Thus, the measured threshold of Aδ-fibre was normalised by rheobase (Figure 4B). The normalised chronaxie was 277 μs (SD of 118 μs). Then, we evaluated the coefficient of variation (CoV) of measured rheobases and its normalised values (Table 1). The CoV is the standard deviation divided by the average value. From Table 1, the CoV for measured value was not significantly affected by pulse width. However, the CoV for the normalised results was smaller than those without normalisation and more variable for short pulse width.

The reaction time of the volunteers is shown in Table 2. The reaction time was stable along the experiment with a maximum variation of 5%, which was consistent with those in previous studies (Kodaira et al., 2014).

**TABLE 2:** Reaction time during sensation threshold detection experiment.

Pulse width (μ s)	Reaction time (s) (Mean ± SD)
60	0.496 ± 0.100
100	0.500 ± 0.103
200	0.519 ± 0.102
400	0.521 ± 0.090
800	0.493 ± 0.085
1600	0.470 ± 0.092

### Model Variability Effect

#### Computational Estimation of Electric Field in the Skin Model

The effect of the needle electrode and stratum corneum layer on the electric field is shown in Table 3. In the skin model, the depth of the needle electrode was varied at 5, 10, 15, 20, and 30 μm from the skin surface, and the stratum corneum thickness was 5, 10, 15, or 20 μm. Considering the characteristics and number of Aδ-fibre, the position of the electric field was selected as *l* = 0–0.35 mm, and the depth at each position was *d_*f*_* = 10–40 μm (steps of 5 μm) based on the ending of the fibre (MacIver and Tanelian, 1993).

**TABLE 3:** Combined effect of the needle depth and thickness of the stratum corneum on the position *l* = 0–0.35 of the electric field (mean value and standard deviation).

Depth of the needle electrode (μm)	Electric field strength (V/m)	
	Mean ± SD	Max
5	3692.3 ± 4023.7	18,195.0
10	3761.6 ± 4190.9	19,824.9
15	3780.9 ± 4236.9	20,927.4
20	3892.2 ± 4525.7	24,322.6
30	4191.2 ± 5407.9	37,029.6

The injection current was set as 20 μA. The induced electric field at the end of the fibre was computed, considering all combinations of the depth of the needle electrode and stratum corneum (total 20 cases). However, the computational result for the models whose electrode depth is 5 μm and stratum corneum is 20 μm was excluded. The reason for this exclusion was computational instability, which is attributable to the stratum corneum thickness; the electric current may not flow with low conductivity of the stratum corneum. Table 3 shows that the stronger the electric field at the end of the fibre, the deeper the needle electrode.

**FIGURE 5:**
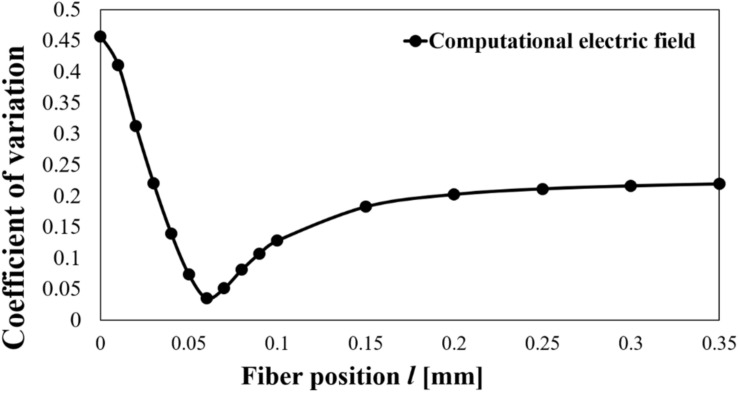
Coefficient of variation on the electric field.

Then, we evaluated the CoV of the electric field strength for all models using the previous parameters (Figure 5). Figure 5 shows that the CoV was more variable close to the needle electrode and minimum value at *l* = 0.06 mm. We varied the fibre diameter *D* at 0.68, 0.80, 1.0, 1.2, or 1.46 μm (Reilly et al., 1997). Assuming that the stimulation threshold of the fibres was inversely proportional to the nerve diameter, the threshold may vary from 0.68 to 1.46 for the abovementioned parameters. The variation of CoV on the position *l* = 0–0.35 mm of computational electric field strength was 0.04–0.46. Considering this combined variability, this may mostly explain the variability of the measured results (CoV) in the rheobase (see Section “Experiment Results of Aδ-Fibre”).

#### Development of Computational *A*δ-Fibre Model

The electrical parameters of nerve activation model were searched so as to coincide with the experimental data. The least squares error between the experimental and computational results was adopted. We changed *C* and *G*_*l*_ for the electrical parameters in the CRRSS model. For the estimated parameters based on the measured results, the corresponding parameters of *C* and *G*_*l*_ are shown in Table 4. The electrical parameters used in the modified CRRSS model are shown in Table 4 (refer to Supplementary Table 1 for more details). Note that the parameters in the CRRSS model were derived from the measurement of the rabbit muscle (Chiu and Ritchie, 1980).

**TABLE 4:** Estimated parameters based on the measured results (see Section “Experiment Results of Aδ-Fibre”).

Parameter	Original parameters	Value		
		*l* = 0.25 mm	*l* = 0.30 mm	*l* = 0.35 mm
Capacity of membrane (*C*)	28.8 nF	26.4 times	20.0 times	12.0 times
Leaked channel conductivity (*G*_*l*_)	128 mS/cm^2^	0.25 times	0.14 times	0.27 times
Rheobase		0.183 mA	0.169 mA	0.161 mA
Chronaxie		247 μs	273 μs	280 μs

**FIGURE 6:**
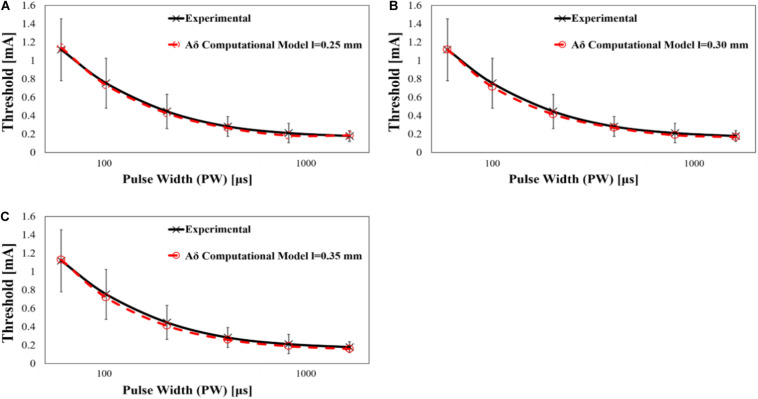
Computed strength–duration curves of Aδ-fibre by single-pulse stimulation (experimental mean value and standard deviation, *n* = 14), **(A)** fibre position *l* = 0.25 mm, **(B)** fibre position *l* = 0.30 mm, and **(C)** fibre position *l* = 0.35 mm.

The computational results of Aδ-fibre are shown in Figure 6, and the parameters of rheobase and chronaxie are shown in Table 4. Also, the rheobase of computed electric field strength at the end of fibre (*d_*f*_* = 10–40 μm) was 262.8–500.4 V/m at *l* = 0.25 mm, 128.9–249.0 V/m at *l* = 0.30 mm, and 66.4–129.4 V/m at *l* = 0.35 mm. The difference between mean experiment and modelled rheobase was 2.98–10.1%. Meanwhile, the difference between the chronaxie values was 1.22–8.52%.

## Discussion

Nociceptive Aδ-fibre is one type of cutaneous sensory neurons. The terminal of Aδ-fibres is found in the epidermis (Kandel et al., 2012). In this study, an experiment for selective stimulation of Aδ-fibre was conducted with the IES targeting them. The activation threshold of Aδ-fibre is characterised by the S–D curve using single-pulse stimulation. Then, an Aδ-fibre model is derived based on experimentally derived S–D curve. The CoV in the measured threshold was more varied for brief pulse injection. One of the possibilities for this is that the ending of the Aδ-fibre is unmyelinated. The difference in the myelin thickness influenced the amount of charge stored in the fibre, so the variation in the short pulse width was large. The chronaxie was estimated in a recent study (Poulsen et al., 2020) as 599–756 μs, which is twice larger than that in our study. However, only four different durations (0.1, 0.5, 1, and 10 ms) were considered in that study. Moreover, our axon model is an extension of the CRRSS model, whereas their own proposal was based on their previous study by Tigerholm et al. (2019).

One difference of this S–D curve was that it was characterised by the injection current, which is not an *in situ* parameter and affected by the stimulator. Then, we conducted computational estimation of electrical parameters of Aδ-fibre based on the CRRSS model. The CoV on the computational electric field where the nerve ending was ranged from 0.04 to 0.45. Combining potential variability of fibre thickness, most experimental variations can be explained. Also, the rheobase value in terms of the internal electric field strength at the fibre terminal (*d_*f*_* = 10–40 μm) ranged from 66.4 to 129.4 V/m at *l* = 0.35 mm. In previous studies, the reported rheobase values were 6.15 V/m using the SENN model (fibre diameter = 20 μm) for electrical peripheral nerve stimulation (Reilly and Diamant, 2011) and 5.9 V/m for magnetic stimulation in the forearm (Havel et al., 1997). In this study, the internal electric field threshold was computed for a fibre diameter model of 1.0 μm, which corresponds, as expected, to a threshold that is inversely proportional to the diameter of the fibre in peripheral nerve fibres.

The original CRRSS model had a chronaxie value 14 times smaller than that of the experimental chronaxie value. Then, we tuned parameters by fitting the chronaxie of computational results in the CRRSS model to that of experimental results. Increasing *C* had an influence on the time to reach the threshold. As a result, when the pulse duration was short, the amount of change in the threshold for pulse duration increased; that is, the chronaxie of computational results increased. In addition, the chronaxie of computational results was simulated in more detail by making *G*_*l*_ small. Therefore, we confirmed that the experimental results could be simulated in more detail based on the conventional CRRSS model.

As we estimate the number of fibres, the computed rheobase was adjusted by changing the fibre position *l* for each diameter *D* (Supplementary Figure 1). The number of fibres was estimated from the fibre position *l*, and the Aδ-fibre density was 160 fibres/mm^2^ (Ebenezer et al., 2007). In the numerical analysis, it is possible that 31.4 to 61.5 fibres (*l* = 0.25–0.35 mm) are stimulated by the IES experiment, while considering the variability of needle depth and depth of the fibres. However, in our investigation, it was not possible to conclude how many fibres are activated by the stimulation; as always, multiple fibres are stimulated. Based on Mouraux et al. (2012), the number of activated Aδ-fibres required to elicit a conscious response was estimated as 59. Therefore, the electrical parameters were searched so as to coincide with the experimental data in fibre position *l* = 0.34 mm (the number of fibres was 59). As a result, the corresponding parameters of *C* and *G*_*l*_ were 13.3 times and 0.34 times, respectively. The rheobase and chronaxie were 0.189 mA and 231 μs, respectively.

Uncertainty in the electromagnetic computational analysis is originated, in large part, from the assignment of electrical conductivity values to each tissue (Motogi et al., 2016). In particular, the reliability of the electric field computation in the stratum corneum is affected by non-linear characteristics of the conductivity for fields exceeding the order of kV/m (Yamamoto and Yamamoto, 1981). We can observe that the field strength could reach the dielectric breakdown, but unlikely owing to the non-linearity of the conductivity, as shown in Figure 5. On the other hand, the influence of the electrode radius was small.

## Data Availability Statement

The raw data supporting the conclusions of this article will be made available by the authors, without undue reservation.

## Ethics Statement

The studies involving human participants were reviewed and approved by the Ethical Committee of Nagoya Institute of Technology. The patients/participants provided their written informed consent to participate in this study.

## Author Contributions

AH and TW conceived and designed the research. TW and ST conducted the experiments. JG-T and ST conducted the experiments and processed the data. All authors analysed the data, wrote the manuscript, and read and approved the manuscript.

## Conflict of Interest

The authors declare that the research was conducted in the absence of any commercial or financial relationships that could be construed as a potential conflict of interest.
