# Supplementary material for: Electrical Characterisation of Aδ-Fibres Based on Human in vivo Electrostimulation Threshold
Source: Front Neurosci. 2021 Jan 5;14:588056. doi: 10.3389/fnins.2020.588056 (PMC7873976; doi:10.3389/fnins.2020.588056)
Supplement: Supplementary file 1 [file Data_Sheet_1.PDF]

Supplementary Material

Supplementary Table 1. The electrical parameters used in the modified CRRSS model.

Parameter	Original parameters	Adjusted for position from needle center *l*		
		l = 0.25 mm	*l* = 0.30 mm	*l* = 0.35 mm
Nernst potential for sodium channels (E_{Na})	115 mV	115 mV	115 mV	115 mV
Nernst potential for leakage channels (E_l)	-0.01 mV	-0.01 mV	-0.01 mV	-0.01 mV
Capacity of membrane at internode ($C_{m,i}$)	28.8 nF	707.7 nF	536.2 nF	321.7 nF
Capacity of membrane at node ($C_{m,n}$)	30.2 nF	796.2 nF	603.2 nF	361.9 nF
Internode membrane resistance ($R_{m,i}$)	218 kΩ	218 kΩ	218 kΩ	218 kΩ
Nodal membrane resistance ($R_{m,n}$)	3.26 kΩ	3.126 kΩ	3.126 kΩ	3.126 kΩ
Myelin conductance (G_m)	26.8 nS	26.8 nS	26.8 nS	26.8 nS
Sodium channel conductance (G_{Na})	1445 mS/cm^2	1445 mS/cm^2	1445 mS/cm^2	1445 mS/cm^2
Leaked channel conductance (G_l)	128 mS/cm^2	32.00 mS/cm^2	17.92 mS/cm^2	34.56 mS/cm^2

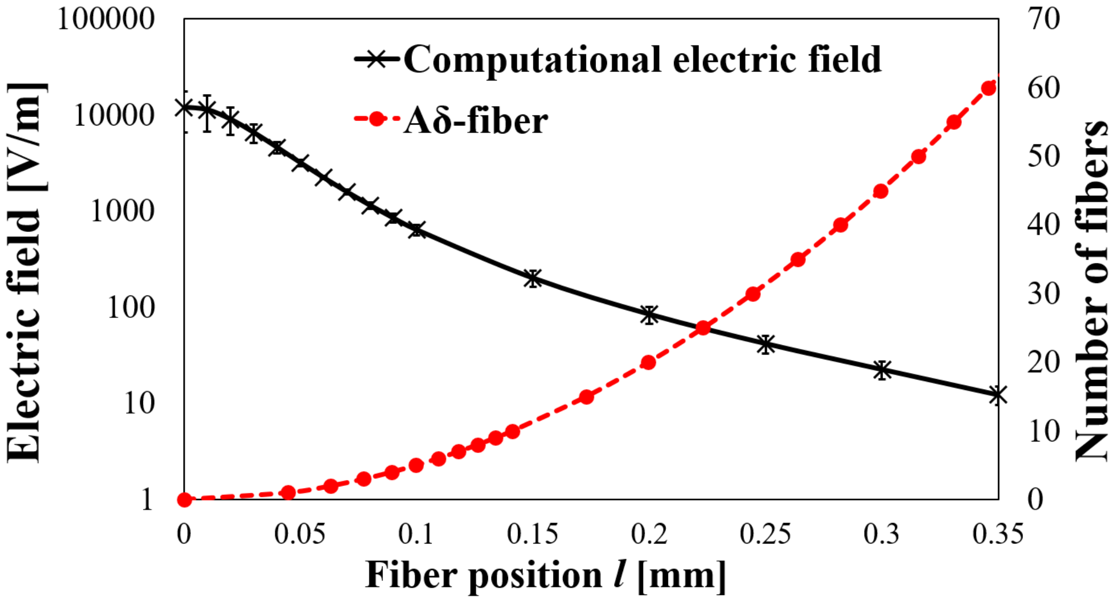

Supplementary Figure 1. Relationship between electric field and number of fibers.
